# Therapeutic Potential of *α*-Synuclein Evolvability for Autosomal Recessive Parkinson's Disease

**DOI:** 10.1155/2021/6318067

**Published:** 2021-11-23

**Authors:** Jianshe Wei, Gilbert Ho, Yoshiki Takamatsu, Eliezer Masliah, Makoto Hashimoto

**Affiliations:** ^1^Tokyo Metropolitan Institute of Medical Science, Tokyo 156-8506, Japan; ^2^Institute for Brain Sciences Research, School of Life Sciences, Henan University, Kaifeng 475004, China; ^3^PCND Neuroscience Research Institute, Poway 92064, CA, USA; ^4^Division of Neurosciences, National Institute on Aging, National Institutes of Health, Bethesda, MD, USA

## Abstract

The majority of Parkinson's disease (PD) is sporadic in elderly and is characterized by *α*-synuclein (*α*S) aggregation and other alterations involving mitochondria, ubiquitin-proteasome, and autophagy. The remaining are familial PD associated with gene mutations of either autosomal dominant or recessive inheritances. However, the former ones are similar to sporadic PD, and the latter ones are accompanied by impaired mitophagy during the reproductive stage. Since no radical therapies are available for PD, the objective of this paper is to discuss a mechanistic role for amyloidogenic evolvability, a putative physiological function of *α*S, among PD subtypes, and the potential relevance to therapy. Presumably, *α*S evolvability might benefit familial PD due to autosomal dominant genes and also sporadic PD during reproduction, which may manifest as neurodegenerative diseases through antagonistic pleiotropy mechanism in aging. Indeed, there are some reports describing that *α*S prevents apoptosis and mitochondrial alteration under the oxidative stress conditions, notwithstanding myriads of papers on the neuropathology of *α*S. Importantly, *β*-synuclein (*β*S), the nonamyloidogenic homologue of *α*S, might buffer against evolvability of *α*S protofibrils associated with neurotoxicity. Finally, it is intriguing to predict that increased *α*S evolvability through suppression of *β*S expression might protect against autosomal recessive PD. Collectively, further studies are warranted to better understand *α*S evolvability in PD pathogenesis, leading to rational therapy development.

## 1. Introduction

**Figure 1:**
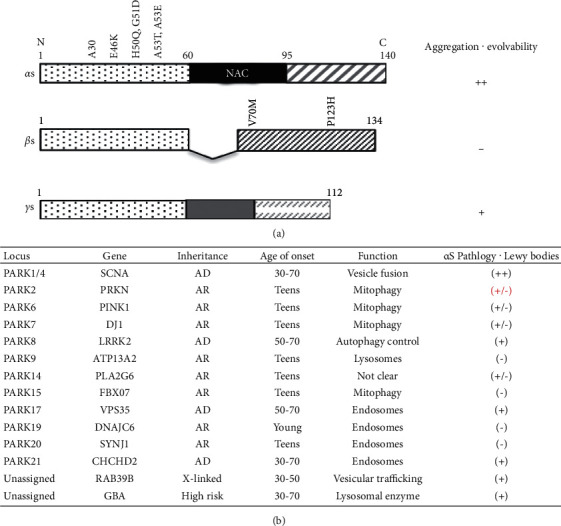
Description of *α*S and other PD risk factors. (a) Diagram of the synuclein family of peptides. *α*S has two related proteins, namely, *β*- and *γ*S. While the N-terminal domains are highly homologous, the C-terminal regions are more divergent. The middle domain of *α*S, referred to as NAC, is highly amyloidogenic, [5], whereas that of *γ*S is somewhat less amyloidogenic. In contrast, the NAC corresponding domain is naturally absent from *β*S. So far, six and two missense mutations have been characterized for *α*- and *β*S, respectively [5]. Since evolvability is supposed to depend on the protofibrillar form of APs, *α*S might exhibit greater evolvability associated with increased aggregation property compared to *γ*S, while *β*S instead may negatively regulate *α*S evolvability through its buffering capacity. (b) Classification of familial PD. Currently, more than twenty familial PD (PARK 1∼21) have been identified, most of which are associated with mutations of either AD- (PARK 1 and 4: SNCA; PARK8: LRRK2; PARK17: VPS35; and PARK 21: DNAJC13) or AR genes (PARK2: Parkin; PARK6: DJ-1; PARK7: PINK1; PARK9: ATP13A2; PARK14: PLA2G6; PARK15: FOXO7; PARK19: DNAJC6; and PARK20: SYNJ1). The former ones are usually late-onset (30–70 years old), frequently during postsenescent aging, whereas the latter ones are early-onset (teens) during the reproductive stage. The functions of the former gene products are related to cellular activity, such as vesicle fusion, mitochondria, autophagy/lysosomes, and endosomes, whereas the latter ones are involved in selective degradation of mitochondria, so called mitophagy. Neuropathologically, the former ones are associated with aggregation of *α*S and formation of Lewy bodies, whereas the *α*S pathologies in the latter are less clear, partially reprinted with modification from Singleton and Hardy (2019) [3] with permission.

Parkinson's disease (PD) represents a set of clinically and pathologically heterogeneous subtypes. The majority (∼85%) of PD is sporadic (sPD) during aging, pathologically characterized by *α*-synuclein (*α*S) aggregation and other cellular dysfunction, involving mitochondria, ubiquitin-proteasome system, and autophagy (Figure 1(a)) [1]. Although the mechanism of sPD remains unclear, it is believed that sPD may be caused by interplay among susceptible genes and environmental factors [2]. In contrast, familial PD is associated with mutations of either autosomal dominant (AD) or autosomal recessive (AR) genes (Figure 1(b)) [3]. However, the neuropathological features of AD-PD are similar to those of sPD, and AR-PD forms are accompanied by impaired mitophagy during reproductive time of life associated with lesser *α*S aggregation [4]. All PD types ultimately lead to the degeneration of dopaminergic neurons in the substantia nigra pars compacta.

Currently, no disease-modifying therapy is available for both familial and sPD, and currently, only symptomatic exists, including oral levodopa [6] and deep brain stimulation [7]. Although transplantation therapy in PD has been extensively investigated using various materials, including human fetal mesencephalic tissue [8] and induced pluripotent stem cells [9], the propagation of *α*S protofibrils might occur from host-to-graft tissues [8]. Furthermore, although *α*S immunotherapy trials in PD are ongoing [10], therapy directed against *α*S aggregation might not be promising given poor and unclear outcomes for amyloid *β* (A*β*) immunotherapy in Alzheimer's disease (AD) [11]. Therefore, it is critical to devise other novel therapeutic strategies.

Despite extensive investigation, a physiological function of amyloidogenic proteins (APs) relevant to neurodegenerative diseases, such as A*β* and *α*S, is unclear. Better understanding of this issue might provide a clue into a new therapy. Based on the similarity with yeast prion, we proposed that evolvability against multiple stressors in the human brain might be related [12,13]. Because AD-PD is similar to sporadic PD in terms of *α*S pathology, while AR-PD is not, the main objective is to discuss that *α*S evolvability might be differentially involved in these subtypes of PD. We speculate that *α*S evolvability might benefit both AD-PD and sPD during development and reproduction, but become detrimental during aging. Furthermore, *α*S evolvability may be regulated by the buffering action of *β*-synuclein (*β*S), a nonamyloidogenic homologue of *α*S [14]. Supposing that increased *α*S evolvability through upregulation of *α*S aggregation during the reproductive portion of the lifespan might be beneficial for AR-PD, suppressing *β*S expression might effectively promote *α*S aggregation/evolvability, being therapeutic in AR-PD.

## 2. Beneficial Aspects of *α*S

*α*S, a member of the synuclein family of peptides that includes two other related proteins—*β*- and *γ*-synuclein (*β*- and *γ*S), was primarily identified as a precursor of the non-A*β* component of AD amyloid (NAC) (Figure 1(a)) [5]. Mounting study revealed that *α*S was neurotoxic. However, if *α*S is simply neurotoxic, why then have such detrimental molecules survived across evolution? Indeed, there are a few studies suggesting that *α*S might also be beneficial.

### 2.1. Physiological Actions of *α*S

Song learning of oscine songbirds in the critical period is one major experimental model of research for learning and memory [15]. In 1992, around the same time when *α*S was isolated as a NAC peptide [5], Clayton and associates identified synelfin through differential hybridization as a gene which is downregulated in the critical period of songbird. Intriguingly, synelfin was the avian homologue of *α*S, which might be essential for bird song memory formation during a critical period in development [16]. Based on this analogy, it is possible that *α*S might play a crucial role for learning and memory during mammalian neurodevelopment [17]. Consistent with this, accumulating evidence suggests that *α*S might be involved in the regulation of synaptic vesicles in a developmentally-regulated manner [18,19]. Furthermore, one may speculate that dementia stimulated by *α*S in aging might be an antagonistic pleiotropy phenomenon of *α*S regulation of memory during development. Antagonistic pleiotropy is a theory of aging in evolutional biology, in which genes that enhance fitness in reproduction but diminish it in aging can be favored by natural selection because selection is stronger early in life compared to later in life [20].

### 2.2. Protective Actions of *α*S

Consistent with this notion, *α*S is shown to cooperate with cysteine string protein a (CSP*α*) in synaptic protection and prevent neurodegeneration [21]. Given that the cochaperone function of CSP*α* is essential for neuronal survival, mice with CSP*α* gene deletion exhibit progressive neurodegeneration. Interestingly, the neurodegenerative phenotype of the CSP*α* mice was ameliorated by cross-breeding with *α*S transgenic mice but was exacerbated by cross-breeding with *α*S mice, suggesting that *α*S may be involved in protection of nerve terminals against injury [21].

**Figure 2:**
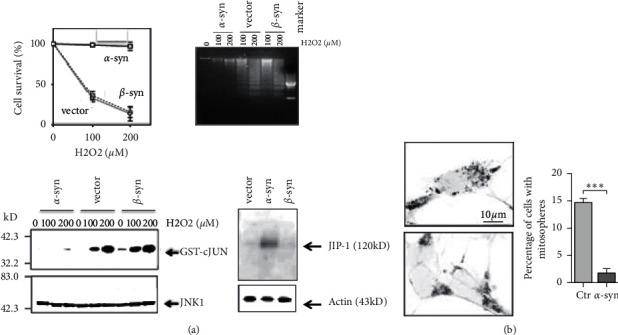
The protective effects of *α*S against oxidative stress in neuronal cells. (a) *α*S is shown to protect against oxidative stress via inactivation of the c-Jun N-terminal kinase stress-signaling pathway in GT1–7 neuronal cells [22]. *α*S-expressing, *β*S-expressing, and vector-transfected cells were treated with hydrogen peroxide (0, 100, 200 *μ*m). At 30 min of treatment, cells were subjected to immunocomplex kinase assay for the assessment of JNK-1 activity. *Note.* This JNK activity is downregulated in the *α*S-overexpressing cells compared to other types of cells. In contrast, immunoblot analysis revealed that expression of JNK-interacting protein (JIP)-1, a JNK-phosphatase, was upregulated in the *α*S-expressing cells. At 24 h, cell survival was determined by the trypan blue exclusion assay and DNA fragmentation assay. Consistent with the results of JNK-1 activity, hydrogen peroxide-treated *β*S-expressing and vector-transfected but not *α*S cells displayed DNA fragmentation, as represented by the laddering of genomic DNA. Reprinted from Hashimoto et al. (2002) [22]. (b) In H4 neuroglioma cells treated with hydrogen peroxide, *α*S was shown to prevent the formation of oxidative stress-induced formation of spherically shaped and hyperpolarized mitochondria, termed “mitospheres,” leading to suppression of apoptosis under the oxidative stress conditions [24]. Reprinted with permission from Menges et al. (2017) [24].

In support of this, a limited number of studies previously showed that *α*S might be involved in the oxidative stress. *α*S was protective against oxidative stress by suppression of the c-Jun N-terminal kinase stress-signaling pathway in GT1-7 mouser neuronal cells (Figure 2(a)) [22]. Similarly, *α*S protected primary cultures of mice cortical neurons from apoptosis by alteration of the MAPK signaling pathway [23]. In addition, it was later shown that *α*S prevented the formation of oxidative stress-induced formation of spherically shaped and hyperpolarized mitochondria, termed “mitospheres,” leading to suppression of apoptosis under the oxidative stress conditions (Figure 2(b)) [24].

## 3. Evolvability of *α*S

As discussed, *α*S may be involved in protection against brain stressors, which is reminiscent of yeast prion. For instance, the [URE3] prion is a nonchromosomal genetic element that produces failure of nitrogen catabolite repression by the self-propagating inactive amyloid form of Ure2p under the nitrogen-deficient condition [25]. Considering that the evolvability of yeast prion is the only physiological phenomenon of APs which is generalizable, where the alteration of aggregation states of APs behave like a genetic switch in response to diverse environmental conditions [13], the concept of yeast prion was applied to APs relevant to neurodegenerative disorders, such as A*β* in AD and *α*S in PD [12].

### 3.1. *α*S Evolvability and sPD

Evolvability is defined as the capacity of a system for adaptive evolution [26]. More specifically, evolvability is composed of two steps: to generate a genetic diversity against environmental conditions including stressors; to deliver their information to offspring [26]. Given that APs including *α*S are intrinsically disordered proteins which might exhibit various forms [27,28], it is assumed that morphologically diverse *α*S protofibrils are formed in a stress-specific manner in response to multiple stressors, such as oxidative stress, kindling, physical stress, and neurotoxicity, and might confer resistance against stressors in parental brain [12]. Among multiple heterogeneous species of *α*S protofibrils, it is predicted that some are toxic, and others are rather beneficial [29]. In support of this notion, it was shown that disordered oligomers were benign to cells, while oligomers with partially formed *β*-sheet cores and highly hydrophobic surfaces were the most inherently toxic species [30]. Furthermore, it was previously characterized that A*β* conferred oxidative stress resistance [31]. Apparently, similar might be the case for *α*S and other APs. Given that some species of APs are protective, it is predicted that the stress resistance of APs might show structure-dependence.

In a prion-like manner, *α*S itself has the capacity to trigger the structural rearrangement of the ubiquitously present *α*S substrate in a self-perpetuating cascade [32]. Following the stress-induced structural alteration of APs into protofibrils, APs might be subjected to transgenerational transmission via germ cells [12,33]. Considering that APs including *α*S are ubiquitously expressed, it is predicted that the prion-like propagation of *α*S might be convenient [28]. Although the heterogeneity of *α*S protofibrils might be beneficial for *α*S evolvability in development/reproduction, *α*-synucleinopathies such as PD might be manifested in parental brain through the antagonistic pleiotropy mechanism in aging [33].

Notably, recent genetic studies, such as genome wide association study, have revealed that the chromosomal genes encoding some molecules relevant to AD-PD, including leucine rich-repeat kinase 2 (LRRK2), vacuolar protein sorting-associated protein 35 (VPS35) [34], and glucocerebrosidase (GBA), might be linked to susceptibility to sporadic PD [3], suggesting that increased *α*S evolvability might be associated with these AD-PD molecules. In particular, the linkage of Gaucher disease to sporadic PD [35] may imply that accumulation of glucocerebroside due to loss of function of GBA may promote *α*S aggregation [36], leading to increased *α*S evolvability. In addition, many environmental causes, including traumatic injury, pesticides, and hypoxia, are recognized in the development of sporadic PD with *α*S aggregation [37–39]. Within our theoretical framework, transmission of such environmental stress information might be beneficial for offspring. Thus, with multiple mechanisms of *α*S aggregation identified, they might all converge at the point of increasing *α*S evolvability.

### 3.2. Increase of *α*S Evolvability in Dominant PD

Since the discovery of A53T *α*S [40], five missense mutations have been identified in SNCA (Figure 1(a)) [41]. In addition, more than 20 genetic loci have been linked to familial PD with mutations of either AD or AR genes (Figure 1(b)) [3]. It has been shown that heterozygous mutations of the AD genes, including SNCA (PARK1and4), LRRK2 (PARK8), VPS35 (PARK17), and CHCHD2 (PARK21), result in various cellular impairments, involving dysfunction of mitochondria, ubiquitin-proteasome system, and autophagy in aging, leading to late-onset PD (Figure 1(b)). Based on the current concept, the increased aggregative properties of *α*S due to AD-PD gene mutations might result in increased *α*S protofibrils transgenerationally transmitted from parent to offspring. Thus, AD-PD molecules may stimulate *α*S evolvability, which might be evolutionarily advantageous. It is also noteworthy that familial dementia with Lewy bodies (DLB) caused by P123H and V70 M mutations of *β*S were characterized paradoxically by *α*S aggregation without aggregation of mutant *β*S (Figure 1(a)) [42]. Presumably, it is possible that structural alterations of *β*S due to missense mutations might promote the formation of *α*S protofibrils, leading to increased *α*S evolvability.

### 3.3. Decrease of *α*S Evolvability in Recessive PD

On the other hand, the significance of *α*S pathology in AR-PD is obscure. In AR-PD, homozygous mutations of recessive genes, such as Parkin (PARK2), DJ-1 (PARK6), PINK1 (PARK7), ATP13A2 (PARK9), PLA2G6 (PARK14), FOXO7 (PARK15), and DNAJC16 (PARK19), result in loss of function of mitophagy, the selective degradation of mitochondria by autophagy, leading to early-onset PD during reproductive life (Figure 1(b)) [3,43,44]. Although it had been believed that AR-PD was not associated with *α*S aggregation [43,45], evidence is accumulating to suggest that *α*S pathologies, including formation of Lewy bodies, are indeed observed in a AR-PD (Figure 1(b)) [46–49]. The precise mechanism of upregulation of *α*S pathology in AR-PD remains elusive. Since homozygous mutation of AR-PD genes results in impairment of mitophagy, a critical cellular function [50, 51], it is possible that *α*S evolvability might be increased by the compensatory mechanism. Therefore, it is predicted that *α*S evolvability may be beneficial for function of mitophagy.

Because of their autosomal recessive nature, carriers are asymptomatic. Consequently, these familial mutations may be not targeted for removal by natural selection. Thus, it is likely that two forms of familial PD may have survived against the pressure of natural selection through distinct mechanisms.

## 4. *β*S Buffering Action on *α*S Evolvability

**Figure 3:**
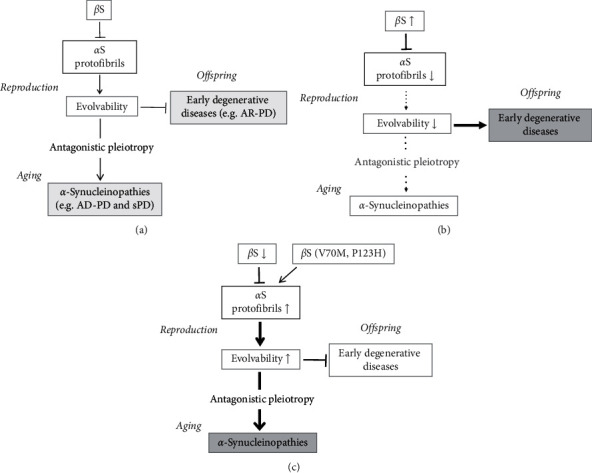
Schematic of the buffering effect of *β*- on *α*S evolvability (a) *α*S protofibrils might be involved in resistance against multiple stresses in parental brains. By virtue of information carried by transgenerational transmission of *α*S protofibrils in reproduction, offspring can cope with forthcoming stresses in the brain, otherwise leading to early degenerative diseases, including AR-PD. On the other hand, *α*-synucleinopathies, such as AD-PD and sPD, are later manifested through antagonistic pleiotropy mechanism in aging. Thus, *α*S evolvability acts as an inheritance of acquired characteristics that are evolutionally beneficial. *β*S might interact with *α*S and inhibit the aggregation of *α*S. As a result, *β*S might mitigate the neurotoxicity of *α*S and negatively regulate *α*S evolvability. (b) If expression of *β*S is too high, the aggregation of *α*S is inhibited and *α*S evolvability would be decreased. Consequently, the brain in offspring cannot obtain stress information enough to escape from developmental diseases. Instead, manifestation of neurodegenerative diseases may be less frequent in aging. (c) If expression of *β*S is too low, the aggregation of *α*S may be stimulated, and *α*S evolvability would be increased. As a result, neurodevelopmental diseases will be suppressed, whereas neurodegenerative diseases may later be manifested through the antagonistic pleiotropy mechanism in aging.

Our view of evolvability relevant to APs in neurodegenerative disorders was initially proposed based on the analogy with evolvability of yeast prion [12]. Accumulating evidence, however, suggests that evolvability of yeast prion might be not beneficial due to its toxicity [52–54], raising a concern that evolvability of APs in human brain might also be the case. In this regard, one possible resolution of this might be by virtue of the buffering role of *β*S on *α*S evolvability (Figure 3).

### 4.1. Is Amyloidogenic Evolvability Beneficial?

The concept of evolvability of yeast prion was created on the notion that the diverse phenotypes conferred by yeast prion, such as [PSI^+^] and [URE3] in response to environmental stressors, which is hereditary to offspring according to cell division, may be a beneficial strategy for yeast thriving in the harsh stressful environment [13]. Several lines of evidence, however, make it clear that the prions might be detrimental to yeast, often lethal [52]. In support of this, even the most mild of the variants of [PSI^+^] and [URE3] prions were detrimental to the host [53,54]. One may assume that the toxicity of amyloidogenic yeast prion might be comparable to the neurotoxicity APs protofibrils in human brain. Similar to yeast prion, APs evolvability in human brain also might not be beneficial.

### 4.2. Regulation of *α*S Evolvability by *β*S

It should be considered, however, that the mode of evolvability might differ significantly between yeast and human brain. The obvious difference is that while yeast proliferates, postmitotic neurons in human brain do not. In yeast, even if yeast prion toxicity is lethal to the majority of the population [53,54], it is predicted that the remaining population could proliferate to compensate. Furthermore, transmission of yeast prion protofibrils to offspring may occur in concert with cell division, a simpler and more efficient means compared to APs in humans that rely on complicated reproductive mechanisms based on germ cells [26]. Thus, even in the presence of cytotoxicity, the evolvability of yeast prion should be more effective compared to APs in human brain.

Also possible, some systems might have evolved to mitigate the toxicity associated with APs evolvability in human. In this regard, it has been described that heat shock protein (HSP) 90 might play a buffering role for evolvability in various biological systems, including plants and drosophila [55,56]. HSPs, however, are commonly expressed between yeast and human biology. Therefore, we presume a possible role for nonamyloidogenic homologues as human-specific modulators of APs evolvability. For instance, *β*S, a member of the synuclein family of peptides, is nonamyloidogenic due to the absence of the amyloidogenic NAC domain (Figure 1(a)) [5]. Given that *β*S not only associates with *α*S but also inhibits *α*S aggregation, leading to suppression of *α*S neurotoxicity [14], it is likely that *β*S might act as a buffer against *α*S evolvability (Figure 3(a)). Consistently, both *α*- and *β*S are abundantly expressed in the central nervous system [57], whereas *γ*S expression is mostly in the peripheral nervous system [58]. Thus, it is possible that the interaction of *α*S with *β*S might be important for evolvability against stressors in the central nervous system, while *γ*S may mainly be involved in evolvability in the peripheral nervous system. Collectively, *β*S could be regarded as “evolution of evolvability,” which is a concept in evolutionary biology that evolution by itself may evolve [59]. The inhibitory effect of *β*S on the aggregation of *α*S has been well studied in transgenic (tg) mice [14,60]. Although it was previously shown that *α*-versus *β*S was upregulated in autopsy brain of DLB [61], the relationship between *α*- and *β*S were never investigated in experimental models. Thus, we focus only on the *β*S actions at the protein level in this paper.

### 4.3. Disease Manifestation due to Disequilibrium of *β*- versus *α*S

**Figure 4:**
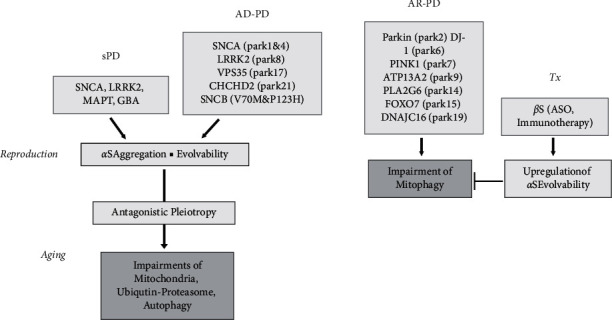
Illustration of the evolvability-based therapeutic strategy against autosomal recessive PD. The majority of PD is late-onset sPD associated with aggregation of a wild type *α*S. sPD might be caused by interaction of various genes, including SNCA, LRRK2, MAPT, and GBA, with environmental factors, such as trauma, pesticide, and hypoxia. In the reproductive stage, *α*S evolvability is beneficial for various cellular functions, such as mitochondria, lysosome-autophagy, and ubiquitin-proteasome system. However, these functions are later impaired through antagonistic pleiotropy, and the late-onset PD is manifested in aging. AD-PD is caused by missense mutations of the AD-PD genes, including SNCA (PARK 1 and 4), LRRK2 (PARK 8), VPS35 (PARK 17), and CHCHD2 (PARK 21) in addition to SNCB (V70 M and P123H). The neuropathology in AD-PD is similar to that of sPD and is characterized by aggregation of *α*S. On the other hand, AR-PD caused by missense mutations of the AD-PD genes, including Parkin (PARK 2), DJ-1 (PARK 6), PINK1 (PARK 7), ATP13A2 (PARK 9), PLA2G6 (PARK 14), FOXO7 (PARK 15), and DNAJC16 (PARK 19), and is associated with impairment of ubiqutin-proteasome, mitochondria, and autophagy, namely, mitophagy in the early-onset PD. Given the buffering effect of *β*S on *α*S evolvability, increase in *α*S evolvability through *β*S downregulation might be therapeutically beneficial in autosomal recessive PD. To achieve this, *β*S expression could be reduced by various methods, including ASO targeting *β*S mRNA and passive *β*S immunization therapy (Tx).

Provided that *α*S evolvability is critical for the development of offspring's brain, the expression of *β*S must be strictly regulated. With markedly elevated *β*S expression, *α*S aggregation is inhibited [14], thus reducing *α*S evolvability. Consequently, offspring's brain cannot obtain sufficient stress information to avoid risk of developmental disorders, and instead, manifestation of neurodegenerative conditions may occur less frequently in aging (Figure 3(b)). In this context, the increased expression of *β*S in dopaminergic neurons might be related to developmental disorders such as autism spectrum disorders (ASD) (Figure 3(b)). Supporting this, it was shown that plasma *α*S levels are significantly lower, while plasma *β*S levels are significantly higher in ASD children than in control individuals [62]. Furthermore, recent study suggests that increased *β*S expression might be relevant to early degenerative diseases such as multiple sclerosis [63]. Thus, it is possible that upregulation of *α*S evolvability by downregulating *β*S might be therapeutically beneficial for early degenerative disorders (Figure 4).

Conversely, markedly diminished *β*S expression promotes *α*S aggregation, increasing *α*S evolvability (Figure 3(c)). This would lead to suppression of neurodevelopmental disorders, whereas neurodegenerative conditions might be increased through the antagonistic pleiotropy mechanism in aging (Figure 3(c)). The mechanism of the decrease of *β*S expression, however, is unclear. As described above, some familial DLB are associated with P123H and V70 M mutations of *β*S without aggregation of mutant *β*S (Figure 1(a)) [42]. Presumably, structural alterations of *β*S due to missense mutations might result in a loss of function of the inhibitory effect of *β*S on *α*S aggregation/protofibrils formation, leading to increased *α*S evolvability [64]. Furthermore, it is also possible that morphological alteration of wild type *β*S may also occur in aging [64]. Notably, it was shown that CSF *β*S concentrations tend to be higher in PD dementia and DLB patients in comparison with PD and controls [65], suggesting that *β*S might be linked to dementia symptoms rather than motor impairment.

Taken together, *β*S may be an important buffer to protect against *α*S neurotoxicity and negatively regulate *α*S evolvability. Considering that *β*S is beneficial for evolution, creation of *β*S may be interpreted as an evolution of amyloid-related evolvability [59]. Yet, the degree of disequilibrium between *β*S and *α*S might underlie both early and late (aging-related) degenerative diseases.

## 5. Therapeutic Strategy Based on Amyloidogenic Evolvability

At present, no effective medical or surgical disease-modifying therapies for PD exist. An exciting prospect, therefore, is that our concept of *α*S evolvability might provide insight into novel therapeutic strategies against PD. Supposing that increase of *α*S evolvability might be beneficial for mitophagy, suppressing expression of *β*S should be therapeutic for recessive PD.

### 5.1. A Therapeutic Strategy against Recessive PD

Given that neurodegeneration in aging might be attributed to amyloidogenic evolvability during reproductive stages through antagonistic pleiotropy [33], the pathological features of *α*-synucleinopathies in aging, involving mitochondria, ubiquitin-proteasome, and autophagy might be attributed to beneficial effects of *α*S on these cellular functions in the developmental/reproductive stage. As described above, *α*S may confer resistance against oxidative stress-induced mitochondrial dysfunction. Yet, the beneficial effect of *α*S on mitochondria in development/reproduction might become detrimental in aging through the antagonistic pleiotropy. A similar mechanism might apply to the effect of *α*S on autophagy. Yet, a number of reports show that *α*S is detrimental to protein degradation systems, such as ubiquitin-proteasome and autophagy, under neurodegenerative conditions in aging [66,67], but there are few studies assessing the effect of *α*S on protein degradation systems during development/reproduction.

It is naturally predicted that upregulating *α*S evolvability might improve or reverse impaired mitophagy in autosomal recessive familial PD. With this in mind, exogenous *α*S might be therapeutically administered to patients, but adverse effects would limit its use. Alternatively, given that *β*S inhibits *α*S aggregation [14], suppressing *β*S expression, such as with antisense oligonucleotides against *β*S mRNA [68] or anti-*β*S immunotherapy [69], might effectively increase *α*S expression, leading to increased evolvability (Figure 4). However, considering that *α*S promotes neurodegeneration in aging, therapeutic dose reduction of *β*S, to be safe and effective, should be applied only during reproductive time of life. Furthermore, caution must be needed to the possibility that increased *α*S expression during reproductive life might underlie neurodegeneration in aging.

Our view of evolvability may explain unresolved issues in the field of PD research. Among numerous familial PD studies in animal models, it is puzzling why there is a lack of apparent neurodegeneration of DA neurons in mouse models of PD [70], including tg mice expressing *α*S in dopaminergic cells [71] and mice with triple knockout of Parkin, PINK1, and DJ-1 [72]. We hypothesize that *α*S evolvability might confer resistance against stressors in dopaminergic cells in mice. Indeed, mouse *α*S aggregates faster than does human *α*S [73], suggesting that the activity of evolvability by *α*S protofibrils in mice may be more potent than that in humans.

### 5.2. Therapeutic Strategy against Dominant PD

In contrast, *α*S aggregation associates with late-onset familial PD with an autosomal dominant inheritance in aging similarly to sPD [74]. Thus, it is generally believed that *β*S may protect against neurodegeneration induced by *α*S protofibrils. Supporting this, neurodegenerative features associated with *α*S transgenic mice were ameliorated in bigenic mice of *α*- and *β*S [14]. Furthermore, the balance of mRNA expression level of *β*- versus *α*S was reduced in autopsy brains of *α*-synucleinopathies [61]. Hence, a differential therapy strategy may be required, i.e., reduce *β*S expression for AR-PD during reproductive time of life, while increasing *β*S expression in AD-PD and sPD in the postreproductive life.

### 5.3. Analogy with Lysosomal Storage Diseases (LSDs)

Notably, the relationship between AD-PD and AR-PD is reminiscent of that between nonneuropathic and neuropathic LSDs such as Gaucher disease (GD). In type 1 GD, neuropathy might be absent by virtue of increase of *α*S evolvability, while PD might be manifested through the antagonistic pleiotropy mechanism in aging. In contrast, neuropathy is severe in early life stage of type 2 and 3 GD due to the decreased *α*S evolvability [75]. It was predicted that increase of *α*S evolvability by suppressing *β*S expression might be potentially therapeutic for type 2 and 3 GD [75]. Thus, the mechanism by which *α*S evolvability differentially involved in either AD or AR PD might be similar to that in GD subtypes.

Besides LSDs, similar differential mechanism might be applicable to the pairs of early and late degenerative diseases. For instance, schizophrenia is an early degenerative disease in development/reproduction stages which might be transgenerationally linked to AD through amyloid evolvability [76]. We assume that increase of *α*S evolvability might be beneficial for schizophrenia, while detrimental to AD. Given the interaction of A*β* with *β*S [77], decreased expression of *β*S might be beneficial for schizophrenia. Furthermore, it is possible that *α*S evolvability might be decreased due to the increased expression of *β*S in ASD in development [62]. Thus, it is tempting to speculate that suppression of *β*S expression might be therapeutic also for ASD.

## 6. Concluding Remarks

In conclusion, *α*S evolvability might be differentially involved between AD-PD and AR-PD. In the former, *α*S evolvability might be beneficial during development and reproduction, but neurodegeneration might be manifested during aging through the antagonistic pleiotropy mechanism. In the latter, the intensity of disease might be severe due to the absence of *α*S evolvability. Since *α*S evolvability is associated with neurotoxicity of amyloid protofibrils in human brain, it is possible that *β*S might act as an important buffer against *α*S evolvability and neurotoxicity. Therefore, *α*- and *β*S, the paired amyloidogenic and nonamyloidogenic homologue, might be have been created through the evolution of evolvability. Such an evolution, however, might have resulted in new disorders, specifically through disequilibrium of *β*- versus *α*S. The relative increase of *β*S might result in downregulation of *α*S evolvability, leading to increased risk of developmental disorders, while conversely, reduced *β*S might upregulate *α*S evolvability, leading to aging-associated neurodegenerative disease.

Admittedly, our scenario linking yeast prion to human brain, germ line, and offspring are not based on solid evidence. However, considering that current medical and surgical therapies for PD, especially AR-PD, are symptomatic and lack significant disease-modifying effects [78], it is intriguing to speculate that increased *α*S evolvability in reproduction might be therapeutic against autosomal recessive familial PD. Given that *β*S inhibits *α*S aggregation, suppression of *β*S expression by various methods, such as ASO and immunotherapy, might effectively increase *α*S expression, producing greater *α*S aggregation and evolvability and leading to amelioration of AR-PD. Notably, the same therapy was applied to LSDs, suggesting that increasing amyloid evolvability might be a common strategy for the treatment of early degenerative diseases. Collectively, further investigation of *α*S evolvability may shed light on new avenues for mechanism-based therapy development in PD.
